# Oxaliplatin, ATR inhibitor and anti-PD-1 antibody combination therapy controls colon carcinoma growth, induces local and systemic changes in the immune compartment, and protects against tumor rechallenge in mice

**DOI:** 10.1136/jitc-2024-010791

**Published:** 2025-03-26

**Authors:** Alexandra Fauvre, Chiara Ursino, Veronique Garambois, Elodie Culerier, Louis-Antoine Milazzo, Nadia Vezzio-Vié, Laura Jeanson, Candice Marchive, Augusto Faria Andrade, Eve Combes, Salima Atis, Gérald Lossaint, François Quenet, Henri-Alexandre Michaud, Lakhdar Khellaf, Ileana Corbeau, Diego Tosi, Nadine Houede, Nathalie Bonnefoy, Olivia Sgarbura, Céline Gongora, Julien Faget

**Affiliations:** 1 Résistance aux traitements et thérapies innovantes, Institut de Recherche en Cancérologie de Montpellier (IRCM), Université de Montpellier (UM), CNRS, Institut Régional du Cancer de Montpellier (ICM), Montpellier, France; 2 Immunity and Cancer Team, Institut de Recherche en Cancérologie de Montpellier (IRCM), Université de Montpellier (UM), Institut Régional du Cancer de Montpellier (ICM), Montpellier, France; 3 McGill University/Research Institute of McGill University, Nada Jabado Lab, Montreal, Quebec, Canada; 4 Institut regional du Cancer de Montpellier, Montpellier, France; 5 Department of Pathology, Montpellier University, Montpellier, France; 6 Medical Oncology Department, Institut régional du Cancer de Montpellier, Montpellier, France; 7 Department of Oncology, University Hospital of Nimes, Nîmes, France; 8 CNRS, Paris, France

**Keywords:** Immune Checkpoint Inhibitor, Colorectal Cancer, T cell, Neutropenia

## Abstract

**Background:**

Colorectal cancer (CRC) is the third most common cancer type and one of the leading causes of cancer-related death worldwide. The treatment of advanced metastatic CRC relies on classical chemotherapy combinations (5-fluorouracil, oxaliplatin or irinotecan). However, their use is limited by the emergence of resistance mechanisms, including to oxaliplatin. In this context, we recently showed that the combination of oxaliplatin and ataxia telangiectasia and Rad3-related protein inhibition (VE-822) is synergistic and may have a potential therapeutic effect in metastatic CRC management.

**Methods:**

In this study, we investigated the role of the VE-822+oxaliplatin (Vox) combination on the immune response and its potential synergy with an anti-programmed-cell Death receptor-1 (PD-1) antibody. We used cell lines and organoids from metastatic CRC to investigate in vitro Vox efficacy and orthotopic syngeneic mouse models of metastatic CRC to assess the efficacy of Vox+anti-PD-1 antibody and identify the involved immune cells.

**Results:**

The Vox+anti-PD-1 antibody combination completely cured tumor-bearing mice and protected them from a rechallenge. Vox was associated with a reduction of tumor-infiltrated neutrophils, CD206^+^ macrophages and regulatory T cells. Vox also induced a deep depletion of blood neutrophils. The increased bone marrow granulopoiesis failed to compensate for the Vox-mediated mature neutrophil depletion. Neutrophil depletion using a mouse recombinant anti-Ly6G antibody partially mimicked the Vox effect on the tumor microenvironment, but to a lower extent compared with the Vox+anti-PD-1 antibody combination. Vox, but not neutrophil depletion, led to the emergence of an Ly6C^+^ PD-1^+^ CD8^+^ T-cell population in the blood and spleen of tumor-harboring mice. These cells were proliferating, and expressed IFN-γ, CD62L, CXCR3 and Eomes. Moreover, the proportion of tumor antigen-specific T cells and of CD122^+^ BCL6^+^ T cells, which shared phenotypic characteristics with stem-like CD8^+^ T cells, was increased in treated mice.

**Conclusions:**

Our work strongly suggests that the Vox+anti-PD-1 antibody combination might significantly improve survival in patients with metastatic and treatment-refractory CRC by acting both on cancer cells and CD8^+^ T cells.

**WHAT IS ALREADY KNOWN ON THIS TOPIC**

- We previously identified ataxia telangiectasia and Rad3-related protein inhibition using VE-822 as a way to restore oxaliplatin sensitivity in colorectal cancer (CRC).

**WHAT THIS STUDY ADDS**

- In this study, we observed that the combination of VE-822+oxaliplatin (Vox) and anti-programmed-cell Death receptor-1 (PD-1) antibody completely cured CRC-bearing mice and protected them from tumor rechallenge that was associated with deep neutrophil biogenesis alteration and the accumulation in the mouse spleen and blood of Ly6C^+^PD-1^+^CD8^+^ T cells sharing phenotypic characteristics with stem cell-like CD8^+^ T cells.

**HOW THIS STUDY MIGHT AFFECT RESEARCH, PRACTICE OR POLICY**

- Our work positions Vox+anti-PD-1 immunotherapy as a new combination strategy to overcome oxaliplatin resistance and improve the survival of patients with metastatic CRC.

## Background

 Colorectal cancer (CRC) is the third most common cancer worldwide and the second leading cause of cancer-related death.^1^ In 2020, the number of cases in Europe was estimated at 500,000, and 15–30% of patients presented synchronous metastases.^2^ The most common metastatic sites are liver (72%), peritoneum (33%) and lung (27%).^2^ Peritoneal carcinomatosis (also called peritoneal metastases, PM) is the most aggressive and most difficult to treat form of metastatic disease and is associated with shorter overall survival.^3^ The management of metastatic CRC is based on various drugs (5-fluorouracil/leucovorin, irinotecan, oxaliplatin, bevacizumab, cetuximab and panitumumab) used in combination.^4^ Immune checkpoint blockade (ICB) with antibodies against programmed-cell death receptor-1 (PD-1) (pembrolizumab and nivolumab)^5^ is proposed to patients with microsatellite instability (MSI; 3–6% of cases). However, drug resistance is a frequent cause of therapy failure, especially in patients with PM.^3^

Oxaliplatin causes interstrand and intrastrand DNA cross-links that block DNA replication and transcription, leading to apoptotic cell death.^6^ It also induces immunogenic cell death (ICD)^7^ that increases cancer cell adjuvanticity and drives the host immune system to mount a stronger antitumor effector function.^8^,^10^ We identified the ataxia telangiectasia and Rad3-related protein (ATR) kinase as a new target involved in oxaliplatin resistance.^11^ ATR is activated in response to replication stress^12^ and recruits partners to prevent genomic instability, promote the continuation of DNA replication and modulate helicase functions, up to the establishment of DNA repair mechanisms.^13^ We demonstrated that the combination of an ATR inhibitor (VE-822) with oxaliplatin (Vox combination) had a synergistic antitumoral effect and might enable better tumor control in patients with oxaliplatin-resistant CRC.^11^

Preclinical observations in vivo suggested that ATR inhibitors, as single agents, can reduce tumor growth in mice,^14 15^ especially when tumors harbor defects in the DNA damage response.^16 17^ Hardaker *et al* evaluated the impact of ceralasertib (an ATR/ATM inhibitor, AZD6738) in syngeneic mouse models of cancer. They reported that the combination of ceralasertib plus anti-programmed-cell death ligand-1 (PD-L1) antibody allowed major tumor control in subcutaneous models of CRC (mouse CT26, MC38 CRC cells) and lymphoma (A20 cells). They found that ceralasertib antitumor activity was dependent on CD8^+^ T lymphocytes and type-I interferon (IFN) signaling.^18^ Importantly, a phase I clinical trial with an ATR inhibitor (PATRIOT) as a single agent in various cancer types showed promising results.^16^ Then, a phase II trial demonstrated that the anti-PD-1 antibody plus ATR inhibitor combination increased overall survival and disease control in patients with lung cancer who did not respond to platinum-based chemotherapy with anti-PD-1 antibody as first-line treatment.^19 20^ Similarly, another phase II study including 31 patients with microsatellite stable gastric cancer who did not respond to first-line I chemotherapy found that anti-PD-1 immunotherapy plus ATR inhibition led to a 22% response rate that was associated with an increase of peripheral blood T-cell receptor-expressing CD8^+^ T cells predicted to have a higher affinity against tumor neoantigens.^21^ The approval of anti-PD-1 treatment for advanced gastric cancer comes from the clinical benefit observed in microsatellite instability and Epstein-Barr virus positive cancer,^22^ highlighting the importance of cancer cell tumor neoantigen load. Interestingly, in this study, patients showing the best response to ATR inhibition plus anti-PD-1 were those having the highest genome mutational burden and default in DNA-repair mechanisms. This might explain why only 22% of the patients showed an objective response.

Altogether, these findings suggest that ATR inhibition may overcome oxaliplatin resistance by acting directly on cancer cells, while simultaneously increasing the antitumor immune response. Therefore, we asked whether the Vox antitumor effect could be enhanced by adding anti-PD-1 immunotherapy. To this aim, we investigated the impact of the Vox plus anti-PD-1 antibody combination in CRC cell lines and tumoroids and systemically in mouse models of CRC-PM.

## Methods

### Patient-derived tumoroids

PM tissue samples were collected during CRS from informed consenting adult patients with CRC-PM in the context of the clinical trial NCT04221464 (BCB COLON, ICM, Montpellier, France) that was promoted by the Institut du Cancer de Montpellier (ICM) . On tissue receipt, tumors were weighed and minced into small fragments with scissors in serum-free RPMI (Gibco 61870–010) with 100 U/mL penicillin-streptomycin (Gibco 15140130). Then, tumor fragments were enzymatically digested using the Human Tumor Dissociation Kit (Miltenyi 130-095-929) and the Miltenyi gentle MACS Dissociator with Program soft 37C- hTDK 1 for mechanical dissociation. Digested samples were passed through 70 µm filters (Miltenyi). Pellets were washed, cells were counted and resuspended in Advanced DMEM/F-12 (Gibco 12634028) with 10 mM Hepes (Gibco 15630056), 2 mM GlutaMAX (Gibco 15630056) and 2 mM penicillin-streptomycin (Gibco 15140130).

Patient-derived tumoroids (PDTOs) were generated by seeding a droplet of 25,000 cells in 5 µL of Matrigel for organoids (Corning 356255) into wells of 96-well black wall clear bottom plates (Greiner 655090) to form domes. After Matrigel polymerization, domes were covered with Advanced DMEM/F-12 (Gibco 12634028) with 10 mM Hepes (Gibco 15630056), 2 mM GlutaMAX (Gibco 15630056), 2 mM penicillin-streptomycin (Gibco 15140130), B-27 Supplement (Gibco 125870010), N-2 Supplement (Gibco 170502048), 1 mM N-acetylcysteine (Merck A9165), 10 µM Y27632 (Selleckchem S1049), 0.5 µM A83-01 (STEMCELL 72024), 10 µM SB202190 (Sigma S7067), 10 mM nicotinamide (Merck N0636), and 50 ng/mL human EGF (Merck E9644). PDTOs were cultured for 5–7 days before treatment (oxaliplatin 0.3 µM, VE-822 1 µM) for 7 days, followed by LIVE/DEAD staining (L3224, Invitrogen) at 37°C for 2 hours prior to imaging with a Celigo Image Cytometer. The live cell area was outlined and quantified with the Celigo confluence application.

### Model of PM initiation

GFP-Luc-CT26-Luc cell suspensions (1.0×10^5^ cells for the first inoculation and 2.0×10^5^ cells for the rechallenge) in 100 µL of serum-free Roswell park memorial institute medium (RPMI) or green fluorescent proteine-luciferase (GFP-Luc)-MC38 cell suspensions (5.0×10^4^ cells for the first inoculation and 1.0×10^5^ cells for the rechallenge) in 100 µL of phosphate-buffered saline (PBS) were injected into the peritoneum in the left iliac fossa of BALB/c mice using a 25-gage needle. When the bioluminescence reached a value between 10^7^ and 10^8^ (~5 days after injection), tumor-bearing mice were randomized and treated with VE-822 (60 mg/kg diluted in NaCl+TPGS) by gavage, oxaliplatin (5 mg/kg diluted in water for injections) or/and anti-PD-1 antibody (5 g/kg diluted in NaCl), or saline solution by intraperitoneal injection, from day 7 for survival studies and from day 12 for immunophenotyping. VE-822 and anti-PD-1 antibodies were injected twice per week, and oxaliplatin once every 2 weeks. Mice were monitored by bioluminescence signal acquisition once per week. Survival curves were constructed following the Kaplan-Meier method. (Ethics Committee approved by the French Ministry, animal facility approval C34-172-27, personal approval (Céline Gongora) 34.142 and protocol approval APAFIS#25 332).

### In vivo experiments rechallenge

For rechallenge experiments, mice cured were second inoculated with twice as many cancer cells as the first time (CT26-Luc cell suspension 2.0×105 cells or MC38-Luc cell 1.0×105). Mice were not treated but monitored by bioluminescence once a week.

### Monitoring and assessment of PM extent

#### Bioluminescence

Once per week, mice were monitored by bioluminescence measurement until the signal reached 10^10^ or day 20 post-graft for immunophenotyping. After intraperitoneal injection of 0.2 mL luciferin (150 mg/mL), 10 min before acquisition, mice were anesthetized in an airtight container with isoflurane and then anesthesia was maintained using a nose cone delivery system in the photon imaging camera (IVIS Lumina II imaging system, Caliper Life Sciences). Images were analyzed with the Living Image V.4.5.2 software after delineation of a region of interest that contained the entire abdominal area of the mouse. The score was the background-subtracted sum of the photon counts in the region of interest.

#### PCI score

Laparotomy was performed and the peritoneal cancer index (PCI) score was determined as in Derrien *et al*.^23^ Briefly, the peritoneal cavity was divided into 13 anatomical regions, and a score from 0 to 3 was given to each region based on the amount and size of peritoneal tumor nodules: 0=no macroscopic tumor; 1=lesion of 1–2 mm, 1–2 sites; 2=lesion of 2–4 mm, 1–2 sites; and 3=lesion >4 mm or >10 sites. The total PCI score was calculated as the sum of the score for each region and ranged from 0 to 39.

#### In vivo neutrophil depletion

The anti-mouse Ly-6G antibody and isotype control were purchased from Bio X Cell. 150 µg of antibody solution was injected intraperitoneally every other day for 6 days.

Dextramer**,** H-2kd/HYLSTQSAL/PE (JE03822 from Imunndex) were used to detect anti-GFP specific CD8^+^ T cells. Briefly, mouse spleen cell suspensions were enriched in CD8^+^ T cells using CD8^+^ T-cell negative selection from Miltenyi (130-104-075) according to provider indications with the MultiMacs 24 cell separator. Then cells were incubated with the Dextramer according to manufacturer indications for 15 min at 4°C. Then an antibody cocktail containing anti-CD8-FITC, anti-CD3-PE-Cy7, anti-CD45-BUV661, anti-Ly6C-BV650 and anti-PD1-PE-Dazzle and viakrome 808 (for references see online supplemental table S1) was added for 15 more minutes. Cells were then washed twice and (paraformaldehyde) PFA fixed as described above before acquisition.

#### Cell sorting

Cell sorting of cancer cells, macrophages, neutrophils and total immune cells from GFP Luc-CT-26 PM-CRC tumor was performed using a BD FACS-Melody by two consecutive sortings on the same samples stained with anti-CD45-perCP-Vio700, anti-297 CD11b-FITC, anti-F4/80-PeCy7, anti-Ly6G-PE and DAPI. Once tumor cell suspension was stained and passed through 70 µM filters, we first sorted GFP^+^CD45^−^ DAPI^−^ cancer cells and CD45^+^ DAPI^−^ immune cells. Once at least 200,000 cells were obtained for each of 300, these two populations, neutrophils (CD45^+^CD11b^high^Ly6G^high^DAPI^−^) and macrophages (CD45^+^CD11b^+^Ly6G^−^F4/80^+^DAPI^−^) until the entire sample was used. Sorting of the four CD8^+^ T-cell subpopulations from blood and spleen was done from a pool of four to five mice repeated five times for each condition. First, CD8^+^ T-cells were enriched using Miltenyi technology as described in the Dextramer staining section. Enriched CD8^+^ T cells from blood or spleen were then stained with anti-CD8-FITC, anti-CD3-PE, anti-CD45-PerCP-Vio700, anti-Ly6C-Pe-Cy7 and anti-PD-1-PEDazzle. We used our four-way FACS-Melody cell sorter to select Ly6C^+^PD-1^+^, Ly6C^−^ PD-1^+^, Ly6C^+^PD1^−^ and Ly6C^−^PD-1^−^ among DAPI^−^ CD45^+^CD3^+^CD8^+^ cells. For the Ly6C^+^PD-1^+^ we usually manage to obtain 50,000 and 250,000 cells from pools of five bloods and spleens respectively in the Vox+anti-PD-1 condition.

## Results

### ATR inhibition combined with oxaliplatin kills cancer cells and reduces PD-L1 expression

**Figure 1:**
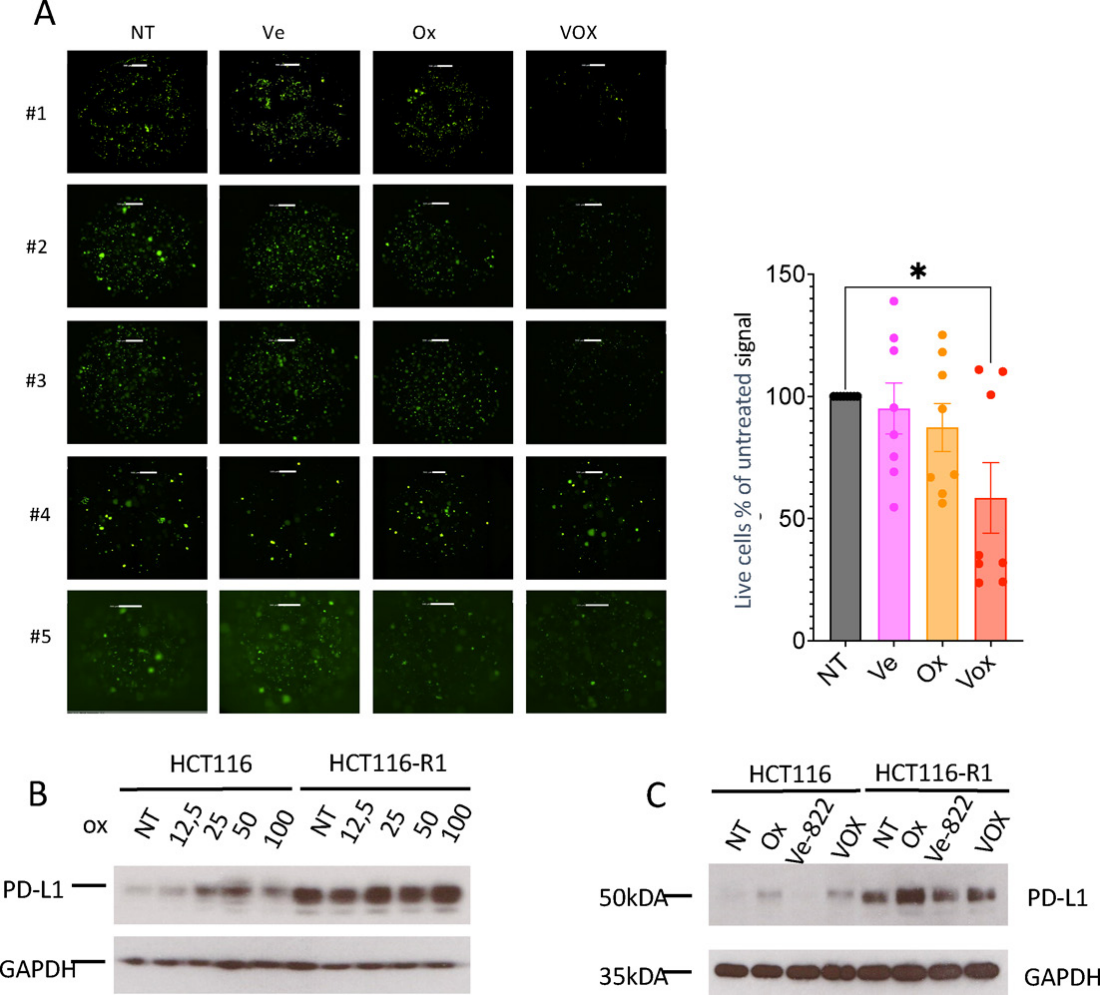
Vox, but not oxaliplatin and VE-822 alone, reduces tumoroid growth. (A) Representative images (left) of patient-derived tumoroids showing viable cells (green) after 14 days of culture in the presence of oxaliplatin (Ox; 0.3 µM), VE-822 (Ve; 1 µM) or Vox (Ox: 0.3 µM+Ve: 1 µM) for 7 days (n=8 patients). Histogram (right) showing the percentage of live cells (green) compared with control (not treated, NT). *p<0.05 (Mann-Whitney test). (B and C): (B) Western blots showing PD-L1 expression in HCT116 and HCT116-R1 cells NT or incubated with increasing concentrations of oxaliplatin (from 12.5 µM to 100 µM) or (C) with oxaliplatin (25 µM), VE-822 (1 µM) or Vox (Ox: 25 µM+Ve: 1 µM) for 24 hours. PD-1, programmed-cell death ligand-1; Vox, VE-822+oxaliplatin.

We hypothesized that the combination of oxaliplatin and the ATR inhibitor VE-822 (Vox thereafter) would offer greater tumor control and immunogenicity, favoring the establishment of a potent antitumor immune response and greater sensitivity to anti-PD-1 antibodies. Therefore, first, we analyzed the impact of exposure to Vox ex vivo on CRC-PM PDTOs. The percentage of living cells decreased significantly in PDTOs exposed to Vox, but not to oxaliplatin or VE-822 alone (figure 1A). As our goal was to determine Vox impact when combined with ICB, we then analyzed PD-L1 expression in parental HCT116 (oxaliplatin-sensitive) and HCT116-R1 (oxaliplatin-resistant) cells.^24^ PD-L1 expression level was significantly higher in HCT116-R1 cells. Moreover, in both cell lines, PD-L1 expression was induced by incubation with oxaliplatin (figure 1B). As ATR might be involved in PD-L1 expression regulation,^25^ we analyzed PD-L1 expression after incubation with oxaliplatin, VE-822 or Vox. VE-822 reduced PD-L1 expression in HCT116 and also in HCT116-R1 cells that displayed stronger basal PD-L1 expression (figure 1C). Moreover, in HCT116-R1 cells, PD-L1 expression was lower in Vox-treated than oxaliplatin-treated cells, suggesting that the ATR inhibitor partially limited oxaliplatin induction of PD-L1 on this cell line.

We concluded that Vox may have different consequences on the antitumor immune response compared with oxaliplatin alone, through cancer cell-dependent mechanisms. This stresses the importance of assessing the impact of ATR inhibition when combined with oxaliplatin and anti-PD-1 antibodies in vivo.

### Vox enhances the response to anti-PD-1 antibodies in CRC-PM mouse models

**Figure 2:**
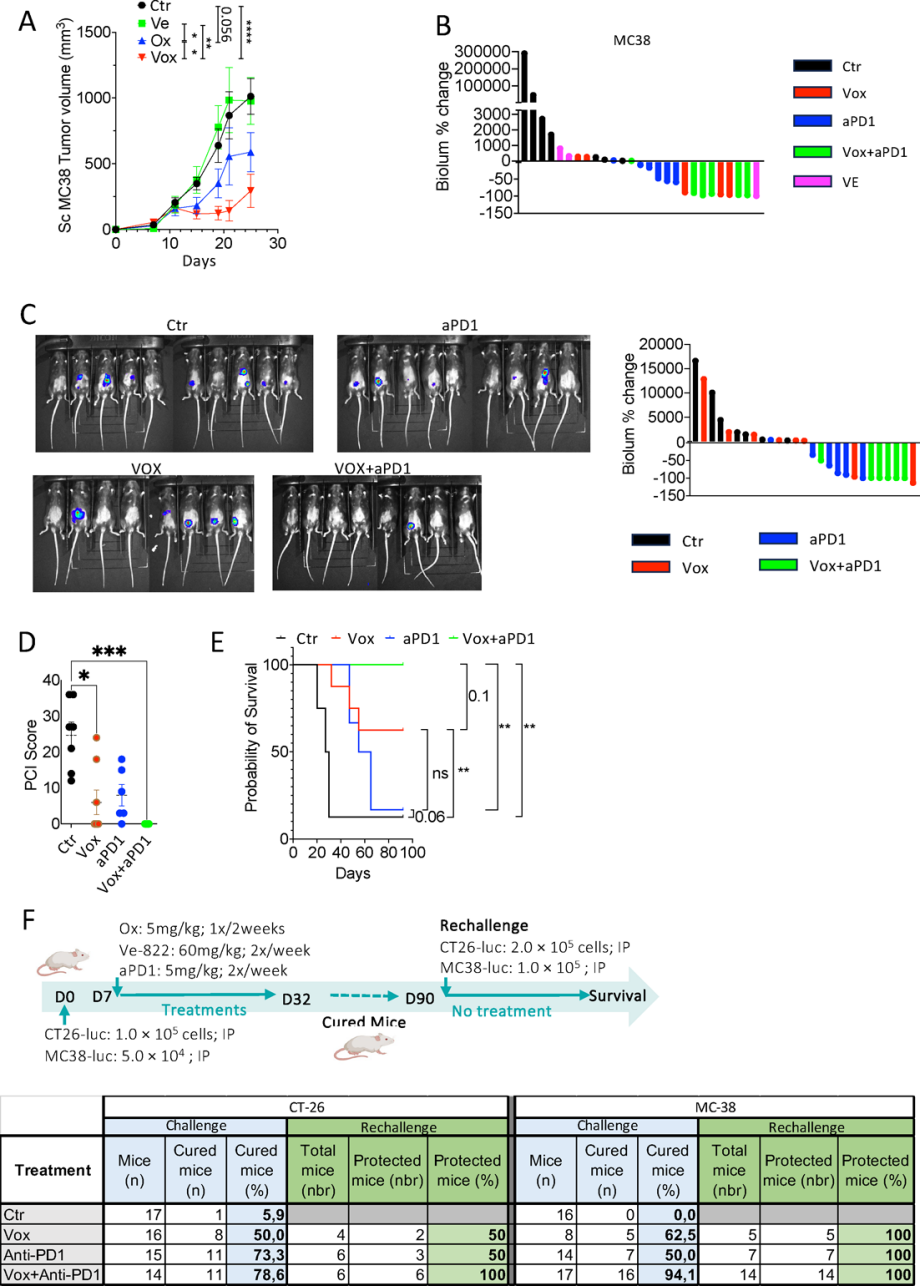
The Vox and anti-PD-1 immunotherapy combination cures mice harboring colorectal cancer-PM. (A) Curves show tumor growth in mice bearing subcutaneous MC38 tumors treated with PBS (NT, n=7), oxaliplatin (Ox; 5 mg/kg once every 2 weeks; n=8), VE-822 (Ve; 40 mg/kg two times a week; n=8) or Vox (same modality as for each drug alone; n=8) for 4 weeks. Mixed model statistic test. (B) Histogram showing the percentage of luciferase signal intensity change in GFP-Luc-MC38 PM-harboring mice treated with PBS (Ctr, n=7), Vox (Ve+Ox, n=6), anti-PD-1 antibody (100 mg two times a week, n=6), VE-822 (Ve; 40 mg/kg, n=2), or Vox+anti-PD-1 antibody (n=6). (C) Representative images (left) of luciferase signal intensity in mice harboring GFP-Luc-CT26 PM (control NT n=9 and Vox+anti-PD-1 antibody n=9) at day 22 after treatment initiation and histogram (right) showing the percentage of luciferase signal intensity change (Ctr n=9, Vox n=6, anti-PD-1 antibody n=6, and Vox+anti-PD-1 antibody n=6). (D) Plot showing the PCI scores of mice harboring GFP-Luc-CT26 PM treated as in C and sacrificed at week 3–8 after treatment initiation. (E) Kaplan-Meier curves showing survival of mice harboring GFP-Luc-CT26 PM treated as in C. (F) Tables showing the number and percentage of tumor-free mice after the first challenge and the number and proportion of cured mice that did not develop tumor lesions following the rechallenge in the indicated conditions. *p<0.05; **p<0.01; ***p<0.001; ****p<0.0001; Ctr, control; ns, not significant; NT, not treated; PBS, phosphate-buffered saline; PCI, peritoneal cancer index; PD-1, programmed-cell death receptor-1; PM, peritoneal metastases; Vox, VE-822+oxaliplatin.

We first treated mice bearing subcutaneous MC38 CRC cell xenografts with oxaliplatin or/and VE-822. Oxaliplatin reduced tumor growth compared with control (Ctr). VE-822 alone did not have any effect on tumor growth; conversely, the combination Vox led to the highest reduction in tumor growth (figure 2A), in agreement with our observations in PDTOs.

As our in vitro data (figure 1C) suggested that VE-822 might interfere with PD-L1 expression in cancer cells, we tested whether Vox could increase anti-PD-1 immunotherapy efficacy in two CRC-PM models. To this aim, we injected intraperitoneally GFP-Luc-MC38 and CT26 CRC cells in syngeneic mice. At day 7, we randomized mice in the following treatment groups: control IgG and PBS (Ctr), Vox, VE-822, anti-PD-1 antibody, and Vox+anti-PD-1 antibody. Luciferase activity monitoring revealed that in both models, the Vox+anti-PD-1 antibody combination led to the strongest tumor regression (figure 2B,C), while Vox or anti-PD-1 antibody alone led to incomplete tumor shrinkage. We confirmed the superiority of the Vox+anti-PD-1 antibody combination by comparing in the GFP-Luc-CT26 CRC-PM model, the tumor burden (PCI score) (figure 2D) and survival (figure 2E) in the different treatment groups.

We then performed a rechallenge experiment in the mice harboring CRC-PM that were cured after administration of the anti-PD-1 antibody or/and Vox. In the GFP-Luc-CT26 model, 94%, 62.5% and 50% of mice were completely cured after treatment with the Vox+anti-PD-1 antibody combination, with Vox, and with the anti-PD-1 antibody, respectively. In the GFP-Luc-MC38 model, 78.6%, 50% and 73.3% of mice were completely cured after treatment with the Vox+anti-PD-1 antibody combination, with Vox, and with the anti-PD-1 antibody, respectively. In the rechallenge experiment, all GFP-Luc-MC38 mice previously cured by the Vox+anti-PD-1 antibody combination were protected against tumor relapse, whereas 50% of those previously cured with Vox or anti-PD-1 antibody died (figure 2F). These experiments show that the Vox+anti-PD-1 antibody combination outperforms each drug alone during the first and also the second challenge. Our results indicate that Vox may increase anti-PD-1 immunotherapy efficacy in terms of tumor regression, and that combining Vox with an anti-PD-1 antibody could promote the induction of a long-term protective immunity against disease recurrence.

To better understand how Vox enhances anti-PD-1 antibody efficacy in vivo, we first assessed in vitro whether oxaliplatin, VE-822 and Vox induced different tumor cell inflammatory signatures. To this aim, we incubated CT26 cells with the three treatments for 24 hours and monitored the transcription of IFN-beta (*Ifn-β*), IFN-stimulated gene-15 (*Isg15*), interleukin-15 (*Il-15*),^26^ interleukin-6 (*Il-6*) and *Cxcl10* that could play a critical role in cytotoxic cell accumulation and inflammation in tumors.^27^ We also analyzed the expression of *Ccl5* and *Ccl2*, two genes encoding chemokines that recruit mostly monocytes/macrophages, myeloid-derived suppressive cells (MDSC) and regulatory T cells (Treg) cells,^28^ as well as C*xcl1*, encoding a chemokine responsible for neutrophil infiltration (for review on chemokines in CRC see^29^). Oxaliplatin and Vox significantly and similarly induced *Ifn-β*, *Il-15* and *Cxcl10*, but not VE-822 alone (online supplemental figure 1). Thus, oxaliplatin induced a chemokine and cytokine response by cancer cells that could be related to the development of an effective antitumor immune control mediated by T and natural killer cells and that was not modified by VE-822 addition. Oxaliplatin also potently induced *Ccl2* and *Cxcl1*. This effect was partially inhibited by the addition of VE-822 (Vox) (online supplemental figure 1). Therefore, the addition of VE-822 (Vox combination) reduced significantly the oxaliplatin-induced upregulation of soluble factors that recruit myeloid cells, such as macrophages (*Ccl5*) and neutrophils (*Cxcl1*), two cell types which can suppress the antitumor immunity. Lastly, Vox led to a strong *Il-6* upregulation compared with oxaliplatin alone (online supplemental figure 1).

Altogether, these results led to the hypothesis that oxaliplatin and Vox might allow the accumulation of antitumor cytotoxic immune cells (*Cxcl10*, *Il-15*, *Ifn-β*). Conversely, ATR inhibition (Vox combination) might prevent oxaliplatin-induced accumulation of potentially immunosuppressive cells by decreasing *Ccl2* and *Cxcl1* expression.

### The ATR inhibitor, oxaliplatin and anti-PD-1 antibody combination leads to a deep reprogramming of the tumor immune compartment

**Figure 3:**
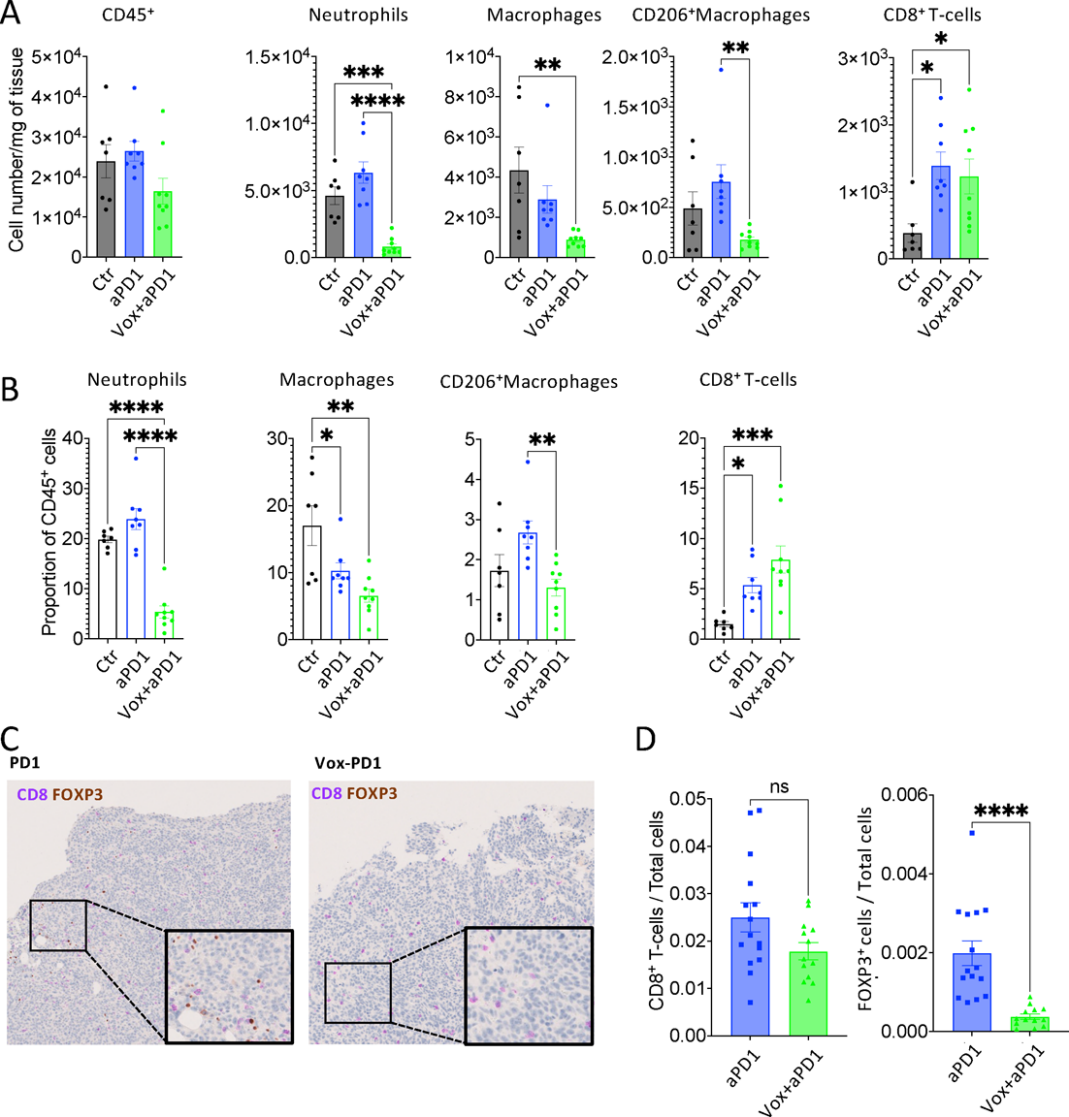
The Vox+anti-PD-1 antibody combination reduces neutrophil, CD206^+^ macrophages and regulatory T cell infiltration in PM nodules. (A) Number per mg of tumor of the indicated immune cell populations measured by quantitative flow cytometry in GFP-Luc-CT26 PM samples harvested from untreated controls (Ctr n=7) and mice treated with the anti-PD-1 antibody (n=8) alone and with Vox (n=9) for 1 week. (B) Histograms showing the percentage among all tumor-infiltrated CD45^+^ immune cells of the indicated immune cell populations from the same experiment described in A. (C) Representative images of CD8 (purple) and FOXP3 (brown) double staining in GFP-Luc-CT26 PM samples from mice treated with the anti-PD-1 alone (n=15) and with Vox (n=13). (D) Histograms showing the ratio of CD8^+^ T cells and FOXP3^+^ cells over the total cell number per tumor from the IHC staining images. *p=0.05; **p=0.01; ***p=0.001; ****p=0.0001; ns, not significant (one-way analysis of variance for A and B and Student’s t-test for D). aPD-1, anti-programmed-cell death receptor-1; Ctr, control; IHC, immunohistochemistry; PM, peritoneal metastases; Vox, VE-822+oxaliplatin.

To better characterize Vox impact on the response to anti-PD-1 immunotherapy, we set-up a quantitative flow cytometry approach to monitor the tumor immune landscape. In mice harboring CRC-PM, the addition of Vox to the anti-PD-1 antibody led to a significant reduction of neutrophils (Ly-6G^+^CD11b^+^CD45^+^), total macrophages (CD11b^+^F4/80^+^) and M2-like macrophages (CD11b^+^F4/80^+^CD206^+^) within the tumor mass, compared with the anti-PD-1 antibody alone (figure 3A). As expected, CD8^+^ T-cell infiltration^30^ in the tumor mass was increased in mice treated with anti-PD-1 antibody and this effect was not changed by Vox addition. Thus, Vox significantly reduced the accumulation of myeloid cells that suppress the antitumor immune response, such as M2-like macrophages, but did not change the massive infiltration by CD8^+^ T cells (the number per mg of tumor) induced by the anti-PD-1 antibody. By quantifying the percentages of the different immune cells in this data set, we confirmed the major decrease of neutrophils and M2-like macrophages and the concomitant increase of CD8^+^ T cells in the tumors of mice treated with the Vox+anti-PD-1 antibody combination (figure 3B).

We then performed immunohistochemistry (IHC) analyses of tumor sections to determine whether the Vox+anti-PD-1 antibody combination changed the spatial distribution of immune cells in the tumor stroma compared with anti-PD-1 antibody alone. We first estimated the size and abundance of tertiary lymphoid structures (TLS) by looking at B-cell (PAX5^+^) and T-cell (CD3^+^) aggregates. We detected many TLS-like structures in almost all tumors without any difference between mice treated with the anti-PD-1 antibody alone and in combination with Vox (not shown). We also co-stained the same samples with antibodies against CD8 and FOXP3. This showed that the proportion of CD8^+^ T cells in the tumor mass was not different between conditions. Conversely, the percentage of FOXP3^+^ Tregs was strongly reduced in the Vox+anti-PD-1 antibody condition compared with anti-PD-1 antibody alone (figure 3C,D). Therefore, we decided to thoroughly investigate the relative impact of ATR inhibition, oxaliplatin and Vox on the tumor immune signature.

### Vox inhibits the accumulation of neutrophils and CD206^+^ macrophages in tumors and upregulates antitumor immune response genes

**Figure 4:**
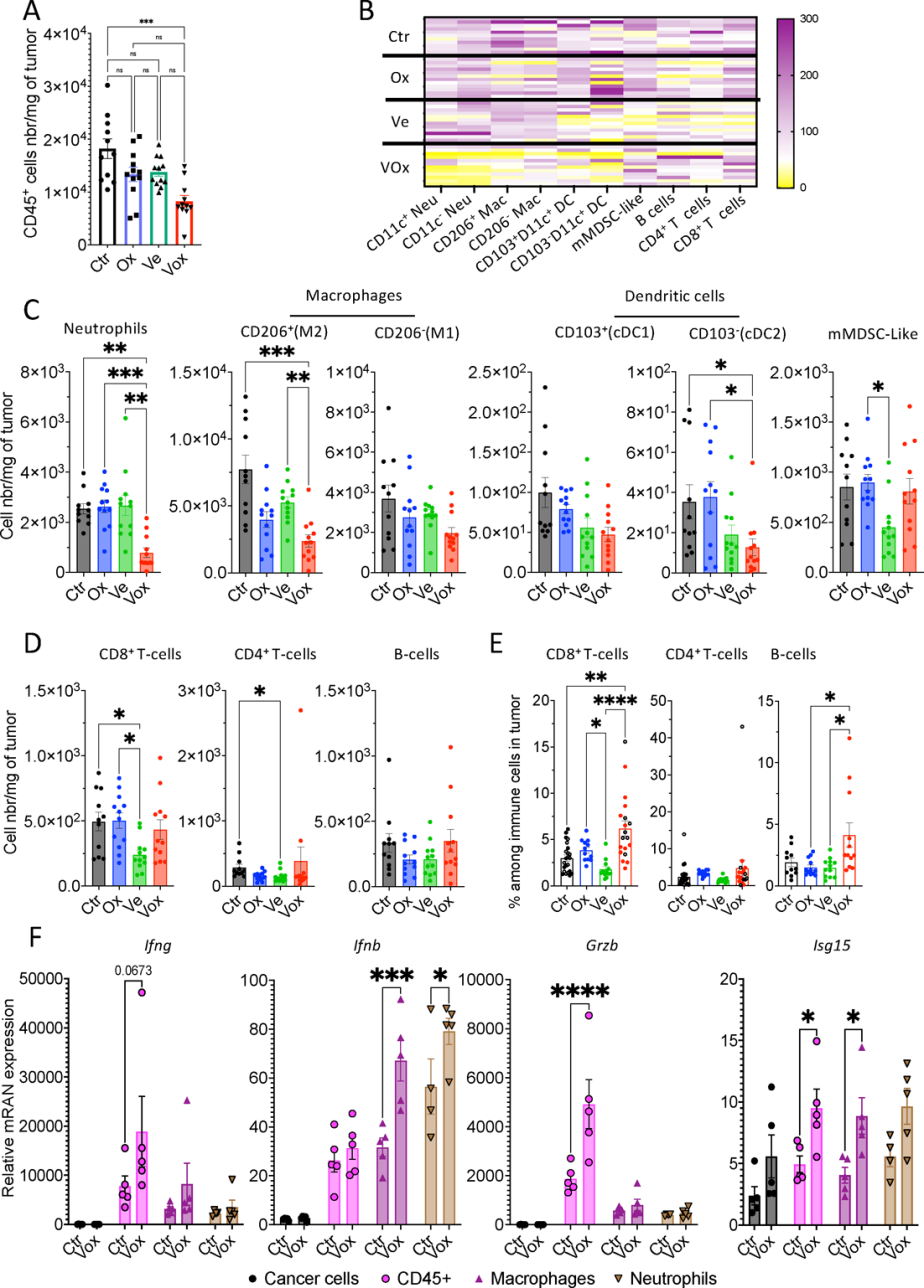
Vox reduces neutrophils and CD206^+^ macrophages and drives the expression of antitumor immune response genes in GFP-Luc-CT26 PM lesions. (A) Number per mg of tumor of CD45^+^ immune cells measured by quantitative flow cytometry in GFP-Luc-CT26 PM samples from untreated control (Ctr n=11) or mice treated with oxaliplatin (Ox n=12), VE-822 (Ve n=12), or Vox (n=12) for 1 week. (B, C and D) Heatmap (B) and histograms (C–D) showing the number per mg of tumor of the indicated immune cell populations measured in the same tumor samples described in (A). (E) Percentages of the indicated immune cell populations relative to all tumor-infiltrated immune cells (CD45^+^) obtained using data from two independent experiments (filled circles: same experiment as in A; empty circles: another experiment where Ctr and Vox-treated PM samples were compared) (Ctr n=25, Ox n=12, Ve n=12, and Vox n=19). (F) Histograms showing the relative mRNA expression in sorted cancer cells (GFP^+^CD45^−^DAPI^−^), total immune cells (CD45^+^DAPI^−^), macrophages (CD45^+^CD11b^+^Ly-6G^−^F4/80^+^DAPI^−^) and neutrophils (CD45^+^CD11b^+^Ly-6G^+^F4/80^−^DAPI^−^) from GFP-Luc-CT26 PM lesions harvested from the control group and from mice treated with Vox for 1 week. Each measurement was obtained from a pool of three mice, n=5 pools per condition. *p=0.05; **p=0.01; ***p=0001; ****p=0.0001; ns, not significant (one-way analysis of variance). Ctr, control; GFP, green fluorescent protein; mRNA, messenger RNA; PM, peritoneal metastases; Vox, VE-822+oxaliplatin.

To better capture the effect of the different treatments on the tumor immune landscape, we performed quantitative flow cytometry analysis of CRC-PM from mice treated with oxaliplatin, VE-822 and Vox. Our gating strategy allows quantifying 10 immune cell populations: CD11c^+/−^ neutrophils (Ly-6G^+^CD11b^+^), CD206^+/−^ macrophages (CD11b^+^F4/80^+^Ly-6G^−^), CD4^+^ or CD8^+^ T cells (CD3^+^Ly-6G^−^F4/80^−^B220^−^), B cells (CD220^+^CD3^−^F4/80^−^Ly-6G^−^), CD103^+/−^ dendritic cells (DC) (CD11b^+^CD11c^+^Ly-6G^−^F4/80^−^CD3^−^B220^−^), and MDSC-like/monocytic cells (CD11b^+^CD11c^low^Ly-6G^−^F4/80^−^CD3^−^B220^−^) (online supplemental figure 2A). Spearman correlation analysis of the different samples showed that lymphoid cells (T and B cells) were negatively correlated with myeloid cells (macrophages, DCs and neutrophils) with the exception of MDSC-like and CD103^+^ cDC1 cells that were positively correlated with CD8^+^ T cells (online supplemental figure 2B). Moreover, Vox, but not oxaliplatin and VE-822 alone, significantly reduced the total number of immune cells per mg of tumor (figure 4A). Vox led to a decrease in the number and percentage of neutrophils (figure 4B,C and online supplemental figure 2C). Moreover, the number and percentage of M2-like (CD206^+^), but not CD206^−^ macrophages, were reduced after treatment with oxaliplatin and particularly with Vox (figure 4C and online supplemental figure 2C). Conversely, CD4^+^ and CD8^+^ T cells (number and percentage) were decreased only in tumors from mice that received VE-822 (figure 4D). In line with the reduction of the myeloid cell proportion, these quantitative modifications translated into higher percentages of lymphocytes among the tumor-infiltrated immune cells (figure 4E). Then, the analysis of PD-L1^+^ cells in each immune cell population showed that only the proportion of PD-L1^+^ CD206^−^ macrophages, CD103^+/−^ DC subsets and MDSC-like cells was increased in tumors treated with oxaliplatin and Vox (online supplemental figure 2D). Thus, our quantitative flow cytometry approach revealed that Vox remodels the tumor immune environment mostly by reducing the number of neutrophils and M2-like macrophages, leading to an increased proportion of CD8^+^ T cells.

We then used flurescence activated cell sorting (FACS) to purify cancer cells (CD45^−^GFP^+^), total immune cells (CD45^+^), neutrophils (CD45^+^Ly-6G^+^CD11b^+^) and macrophages (CD45^+^F4/80^+^CD11b^+^) from CT26-GFP-Luc PM from control and Vox-treated mice to evaluate the expression of tumor-immunity-related genes in the different cell populations. In Vox-treated tumors, *Ifn-b* was upregulated in neutrophils and macrophages and *Ifn-g* (almost significant), *Isg15* and *Grzb* in total immune cells. Conversely, *Ptprc* (CD45) expression was similar in the control and Vox conditions (figure 4F and online supplemental figure 2E). These results suggest that Vox induces a strong antitumor immune response through IFN signaling and cytotoxic cells.

### Vox blocks neutrophil biogenesis in the bone marrow and neutrophil depletion partially mimics the Vox effect on the tumor immune ecosystem

As our findings showed that Vox strongly reduced neutrophils in tumors, we analyzed the expression of *Cxcl1*, *Cxcl2* and *Cxcl5* (genes encoding neutrophil-recruiting chemokines) in FACS-sorted cancer cells, total immune cells, neutrophils and macrophages. Compared with control, Vox led to a slight decrease (not significant) of *Cxcl1* expression in cancer cells, to a strong downregulation of *Cxcl5* in total immune cells and macrophages, and to a modest upregulation of *Cxcl2* in tumor macrophages (online supplemental figure 3A). We concluded that the Vox effect on neutrophil infiltration was likely due to a deep alteration of the neutrophil pool.

**Figure 5:**
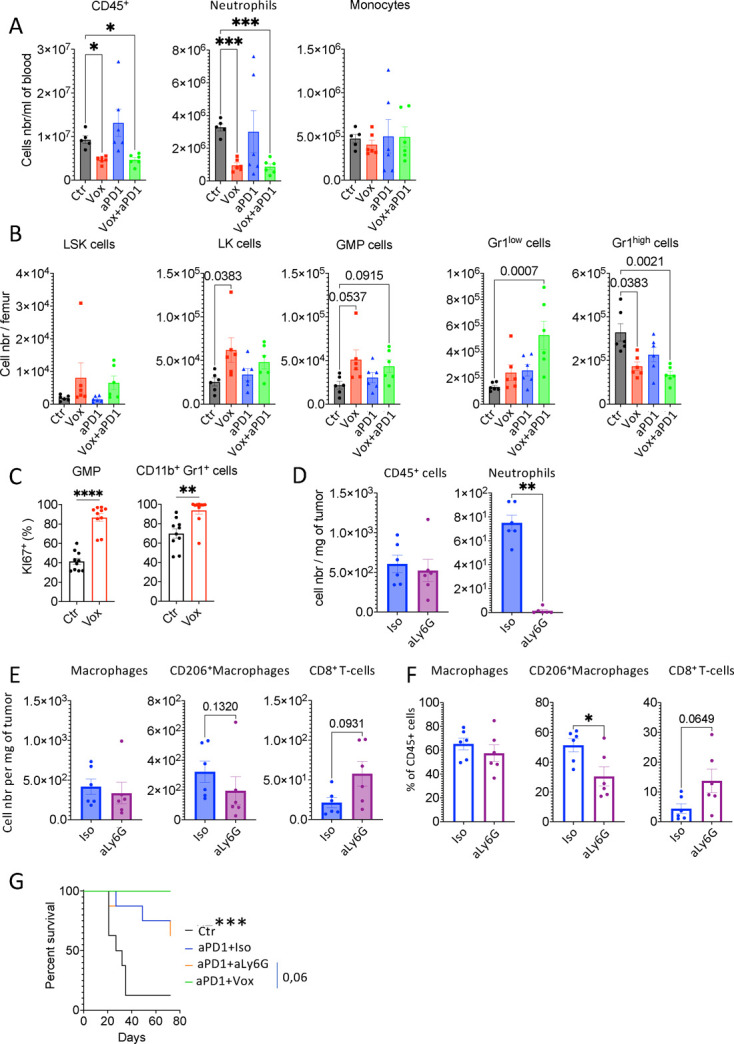
Vox alters neutrophil biogenesis, but neutrophil depletion does not recapitulate Vox antitumor effect. (A) Number per mL of blood of the indicated immune cell populations measured by quantitative flow cytometry in GFP-Luc-CT26 PM mice that were not treated (Ctr n=5) or treated with Vox (n=6), anti-PD-1 antibody (n=6), or Vox+anti-PD-1 antibody (n=6) for 1 week. (B) Number per femur of hematopoietic stem cells (LSK), immature lineage-negative c-Kit+ (LK) cells, granulocyte/monocyte progenitors (GMP), CD11b^+^Gr-1^low^ and differentiated CD11b^+^Gr-1^high^ neutrophils from the same mice as in A. (C) Ki67 expression in GMPs and differentiated neutrophils (CD11b^+^Gr-1^+^) in the control condition (Ctr n=10) or after Vox treatment (n=10) in mice bearing GFP-Luc-CT26 PM. (D) Total CD45^+^ cells and Ly-6C-CD11b^+^ neutrophils by quantitative flow cytometry in GFP-Luc-CT26 PM lesions from mice treated with a control antibody (Ctr) or 150 mg of mouse recombinant anti-Ly-6G antibody (n=6 per condition). (E and F) Cell number per mg of tumor tissue (E) and percentage relative to all tumor-infiltrated immune cells (F) of the indicated immune cell populations from the same samples as in D. *p=0.05; **p=0.01; ***p=0.001; ****p=0.0001 (one-way analysis of variance). (G) Kaplan-Meier survival curves of mice treated with isotype IgG control (not treated, n=8), anti-PD-1 antibody plus isotype IgG control (PD-1+IsoT, n=8), anti-PD-1 antibody+anti-Ly-6G antibody (n=8) and anti-PD-1 antibody+Vox (n=8); ***p=0.001. aPD-1, anti-programmed-cell death receptor-1; Ctr, control; GFP, green fluorescent protein; PM, peritoneal metastases; Vox, VE-822+oxaliplatin.

Consistently, circulating blood immune cells, especially neutrophils, were significantly decreased in mice treated with Vox or Vox+anti-PD-1 antibody (figure 5A). Then, to determine whether Vox directly acted on hematopoietic stem and progenitor cells, we designed a quantitative flow cytometry panel that allows identifying mature (Gr-1^hi^CD11b^+^) and immature (Gr-1^hi^CD11b^low/neg^) neutrophils, granulocyte/monocyte precursors (GMP) (Lin^neg^CD11b^neg^cKit^+^SCA1^neg^CD16/32^+^CD34^+^) and hematopoietic stem cells (LSK cells) (Lin^neg^CD11b^neg^cKit^+^SCA1^+^) in bone marrow samples from mice harboring CRC-PM and treated with the different drugs (online supplemental figure 3B). Vox tended (not significant) to increase the number of LSK and GMP cells in the bone marrow and significantly reduced the number of Gr-1^hi^CD11b^+^ mature neutrophils (figure 5B). Addition of an anti-Ki67 antibody in our flow cytometry panel revealed that the percentage of proliferating GMPs was increased after treatment with Vox (figure 5C). These data suggested that Vox might block cell transition from GMPs to mature neutrophils in the bone marrow. As neutrophil biogenesis is partially regulated by neutrophil abundance and aging,^31^ our results suggest that the Vox negative effect on neutrophil biogenesis could lead to an increased retention of stem cells in the hematopoietic niches and to enhanced GMP proliferation, making them a preferential Vox target (online supplemental figure 3C).

To determine whether neutrophil loss could be responsible for the Vox-mediated tumor immune landscape reprogramming, we performed neutrophil depletion in the GFP-Luc-CT26 PM model using an IgG2a murinized anti-Ly-6G antibody derived from the 1A8 clone. This strategy, recently described by Olofsen *et al*,^32^ led to a stable and deep depletion of tumor and blood neutrophils identified as Ly-6C^int^CD11b^+^ cells (figure 5D and online supplemental figure 3D). Following neutrophil depletion, the number and percentage of CD206^+^ M2-like macrophages were decreased, whereas the number and percentage of CD8^+^ T cells were increased in tumors (figure 5E,F). As neutrophil depletion seemed to mimic Vox impact on the tumor immune cell environment, we asked whether it could also enhance the anti-PD-1 antibody efficacy. The Vox+anti-PD-1 antibody combination allowed for the cure of 100% of mice (n=8) harboring GFP-Luc-CT26 PM. Three of the eight mice treated with the anti-Ly-6G+anti-PD-1 antibody combination died as observed with the anti-PD-1 antibody alone (figure 5G). This suggests that although neutrophil depletion partially mimics the Vox effect on the tumor immune cell environment, this is not the mechanism through which Vox sensitizes tumors to anti-PD-1 immunotherapy.

### The Vox+anti-PD-1 antibody combination leads to the emergence of PD-1^+^Ly-6C^+^CD8^+^ T cells

**Figure 6:**
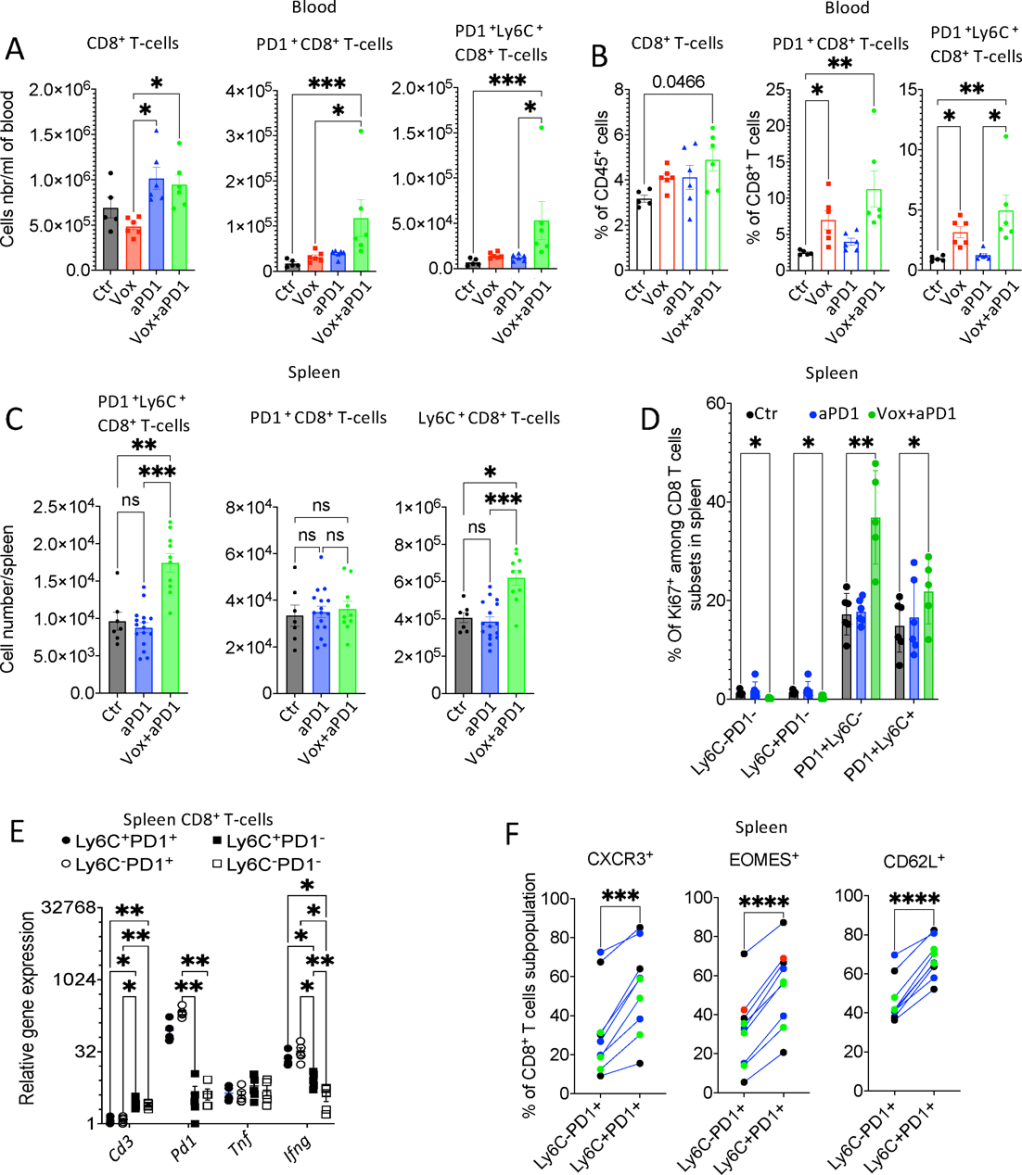
Vox treatment leads to the emergence of Ly-6C^+^PD-1^+^ CD8^+^ T cells in blood and spleen. (A and B) Number per mL of blood (A) and percentage relative to immune cells (B) of the indicated CD8^+^ T-cell populations from GFP-Luc-CT26 PM mice untreated (Ctr n=5), or treated with Vox (n=6), anti-PD-1 antibody (n=6) and Vox+anti-PD-1 antibody (n=5). (C) Number per spleen of CD8^+^ T cells with the indicated phenotypes from GFP-Luc-CT26 PM mice untreated (Ctr n=7), or treated with the anti-PD-1 antibody (n=16) or with Vox+anti-PD-1 antibody (n=10). (D) Histogram showing the percentage of Ki67^+^ cells among the indicated CD8^+^ T-cell subpopulations from GFP-Luc-CT26 PM mice untreated (Ctr n=6) or treated with the anti-PD-1 antibody (n=6) or Vox+anti-PD-1 antibody (n=5). (E) Relative messenger RNA expression of the indicated genes in the indicated CD8^+^ T-cell subpopulations purified from the spleen of Vox-treated GFP-Luc-CT26 PM mice (n=5). (F) CXCR3, Eomes and CD62L expression in CD8^+^ T cells (flow cytometry analysis) from the spleen of Vox-treated GFP-Luc-CT26 PM mice. *p=0.05; **p=0.01; ***p=0.001; ****p=0.0001; ns, not significant (one-way analysis of variance). aPD-1, anti-programmed-cell death receptor-1; Ctr, control; GFP, green fluorescent protein; PM, peritoneal metastases; Vox, VE-822+oxaliplatin.

By comparing blood cells in mice treated with Vox and Vox+anti-PD-1 antibody we observed that besides neutrophil reduction, these drugs led to an increase of PD-1^+^CD8^+^ T cells and of PD-1^+^Ly-6C^+^CD8^+^ T cells, particularly in the Vox+anti-PD-1 antibody combination (figure 6A). Furthermore, in the circulating CD8^+^ T-cell population, the increased percentage of PD-1^+^ cells on Vox treatment translated into a higher proportion of Ly-6C^+^PD-1^+^ double positive cells (figure 6B). We also detected a higher number of Ly-6C^+^PD-1^+^ double-positive CD8^+^ T cells in the spleen of Vox+anti-PD-1 antibody-treated mice compared with anti-PD-1 antibody-treated mice (figure 6C). Unlike in blood samples, in the spleen, the percentage of PD-1^+^ cells was similar in all conditions, whereas the number of Ly-6C^+^ cells was increased in the Vox+anti-PD-1 treated group (figure 6C). In tumors, the number of Ly-6C^+^PD-1^+^ double-positive CD8^+^ T cells was slightly higher in the Vox+anti-PD-1 antibody group than in untreated controls (online supplemental figure 4A), and their percentage among CD8^+^ T cells was higher in the Vox+anti-PD-1 antibody group than in the anti-PD-1 antibody group (online supplemental figure 4B). This suggests that this effect is specifically induced by the Vox+anti-PD-1 antibody combination because neutrophil depletion (combined or not with the anti-PD-1 antibody) did not increase the number/proportion of Ly-6C^+^PD-1^+^ double-positive CD8^+^ T cells in the blood (online supplemental figure 4C).

In the spleen, analysis of Ki67 expression in the different CD8^+^ T-cell subsets showed that despite PD-1 expression, Ly-6C^+^PD-1^+^ double-positive CD8^+^ T cells were proliferating more compared with Ly-6C^−^PD-1^−^ and Ly-6C^+^PD-1^−^ cells and their percentage was increased after treatment with the Vox+anti-PD-1 antibody combination (figure 6D). We purified these four subpopulations and performed gene expression analyses. *Ifn-g,* but not *Tnf,* was upregulated in PD-1^+^ CD8^+^ T cells from spleen and blood from Vox+anti-PD-1 treated mice (figure 6E and online supplemental figure 4D). Moreover, in the spleen, the expression levels of CXCR3, Eomes and CD62L were increased in Ly-6C^+^PD-1^+^ double-positive CD8^+^ T cells compared with PD-1^+^Ly-6C^−^ cells (figure 6F), suggesting that these double-positive cells might have a central memory phenotype in the spleen, consistent with a stem-like phenotype.^33^

### The Vox+anti-PD-1 antibody combination protects cured mice against tumor rechallenge possibly by inducing stem-like Ly-6C^+^ CD8^+^ T cells

**Figure 7:**
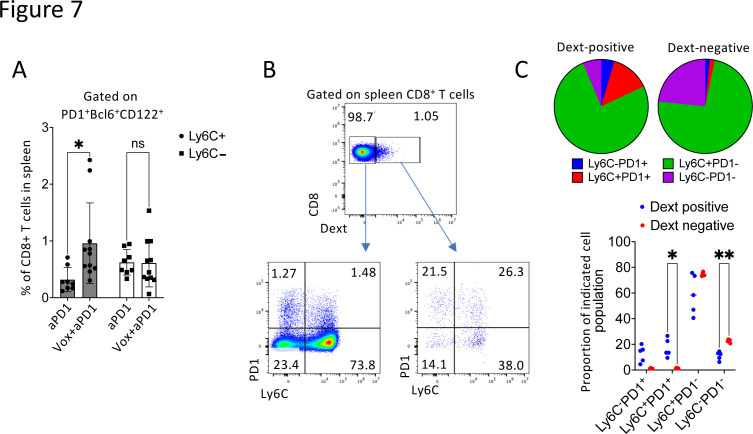
The Ly-6C^+^PD-1^+^ CD8^+^ T-cell population contains a strong proportion of tumor antigen-specific T cells. (A) Histogram showing the percentage of BCL6^+^CD122^+^ cells among PD-1^+^Ly-6C^+^ T cells (black) and PD-1^+^Ly-6C^−^ CD8^+^ T cells (white) from the spleen of GFP-Luc-CT26 PM mice treated with the anti-PD-1 antibody (n=8) or Vox+anti-PD-1 antibody (n=11). (B) Plots showing the gating strategy to identify GFP-specific T-cell receptor-positive cells after Dextramer staining and Ly-6C^+^ and PD-1^+^ cells among them in the spleen of Vox+anti-PD-1 antibody-treated GFP-Luc-CT26 PM mice. (C) Pie chart (top) and histogram (bottom) showing the distribution of dextran-positive (right) and dextran-negative (left) cells in the different CD8^+^ T-cell subpopulations from the spleen of Vox+anti-PD-1 antibody-treated GFP-Luc-CT26 PM mice (n=5). *p=0.05; **p=0.01 (one-way analysis of variance). PD-1, anti-programmed-cell death receptor-1; GFP, green fluorescent protein; PM, peritoneal metastases; Vox, VE-822+oxaliplatin.

To determine whether Ly-6C^+^PD-1^+^ double positive CD8^+^ T cells have stem-like properties, we analyzed the expression of *Bcl6*, *Tcf1/7* and *Lef1* in Ly-6C^+^PD-1^+^ and Ly-6C^−^PD-1^+^ CD8^+^ T cells purified from spleen and blood samples of Vox+anti-PD-1 antibody-treated mice. In the spleen, their expression levels were not different between T-cell subpopulations (online supplemental figure 5A). Conversely, in blood, *Bcl6* mRNA expression was higher in Ly-6C^+^PD-1^+^ double-positive cells (online supplemental figure 5B). Flow cytometry analysis of CD122 and BLC6 expression on these cells from spleen showed that Vox+anti-PD-1 antibody led to an increase of Ly-6C^+^PD-1^+^CD122^+^BCL6^+^ cells among CD8^+^ T cells compared with anti-PD-1 antibody alone (figure 7A, online supplemental figure 5C,D). Although these cells shared many characteristics with stem-like CD8^+^ T cells, we could not detect *Tcf1* expression in any of the PD-1^+^ subpopulations from spleen and blood (not shown). However, by identifying tumor antigen-specific T cells (GFP as tumor antigen) by Dextramer staining, we could investigate PD-1 and Ly-6C expression in these cells in spleen samples. Tumor antigen-specific T cells included a strong proportion of Ly-6C^+^PD-1^+^ double-positive cells compared with Dextramer-negative T cells (figure 7B,C).

Altogether, these results led us to hypothesize that the Vox+anti-PD-1 antibody combination leads to the emergence of Ly-6C^+^PD-1^+^ double-positive CD8^+^ T cells in the spleen. This population includes a strong proportion of tumor antigen-specific cells and shares phenotypic characteristics with stem-like CD8^+^ T cells. Therefore, the Vox+anti-PD-1 antibody combination allows for deep tumor control and could also establish long-term protection against tumor recurrence (see figure 2).

We concluded that ATR inhibition in combination with oxaliplatin and anti-PD-1 immunotherapy improves tumor control and also provides long-term protection against disease recurrence. This effect may be explained by a stronger induction of Ly-6C^+^PD-1^+^ lymphocytes, which share phenotypic similarities with stem-like CD8^+^ T cells.

## Discussion

We decided to investigate the impact of ATR inhibitor and oxaliplatin in CRC-subcutaneous models to confirm previous results.^11^ Then we investigated the effect of these drugs in peritoneal carcinomatosis through intraperitoneal cancer cell transplantation, knowing that the tumor microenvironment might be substantially different. We found that in CRC-PM mouse models, ATR inhibition in combination with oxaliplatin and anti-PD-1 immunotherapy completely cures and also protects against tumor rechallenge.

This finding in preclinical models is of major importance and may be soon translated into clinical practices. Indeed, several ATR inhibitors are tested in clinical trials (online supplemental table S3). We identified 47 clinical trials (23 phase I and 24 phase I/II or II) on ATR inhibitors: tuvusertib/M1774 (Merk) (n=12 trials), ceralasertib/AZD6738 (AstraZeneca) (n=9 trials), berzosertib/M6620/VE-822 (Merk) (n=9 trials), and elimusertib/BAY1895344 (Bayer) (n=9 trials). Most of these clinical trials are in patients with metastatic and treatment-refractory solid tumors. ATR inhibition is combined with Poly(ADP-ribose) polymerase (PARP)-inhibitors (niraparib, veliparib or olaparib) in 11 trials, with genotoxic agents (gemcitabine, carboplatin, paclitaxel, lurbinectedin, topotecan, irinotecan or irinotecan+5-fluorouracil, + leucovorin (FOLFIRI)) in 14 trials, and with anti-PD-(L)1 ICB (durvalumab, pembrolizumab, avelumab, cemiplimab) in 10 trials. These clinical trials highlight the idea that ATR inhibition can be used to overcome resistance to chemotherapy and to sensitize tumors to ICB.

ATR inhibitors and anti-PD-1 immunotherapy have been associated with radiotherapy in hepatocellular carcinoma and CRC preclinical models. In both cases, triple therapy resulted in a great improvement in terms of tumor growth control and survival.^34 35^ Interestingly, in CRC models, the benefit of the triple therapy was evident both in MSI and in microsatellite stable diseases.^34^ Furthermore, triple therapy treated mice had an increased percentage of central memory and effector memory CD8 T cells in the tumor microenvironment and increased CD8^+^ T effector memory and CD4 central memory T cells in spleen compared with radio-immunotherapy. Thus, the addition of ATR-inhibitor to standards of care treatments, including ICB, was shown to enhance the antitumor T-cell response, rising a very specific effect of ATR-inhibitor on the immune compartment on top of its inhibitory impact on the cancer cell DNA-repair machinery.

In our study, we evaluated a novel combination integrating ATR inhibition, oxaliplatin and anti-PD-1 ICB. We used models of CRC-PM, a pathology for which we did not identify any ongoing clinical trial involving ATR inhibition. Our promising preclinical findings should lead to the design of clinical trials to assess the Vox+anti-PD-1 ICB regimen in patients with metastatic oxaliplatin-resistant CRC who have a very low survival rate.^3 36^

Our analysis of the mechanisms through which Vox might increase ICB efficacy showed that Vox reduces tumor infiltration by neutrophils and CD206^+^ macrophages. Regarding neutrophil biogenesis, similar observations were previously reported in mice treated with an ATR inhibitor and anti-PD-1 ICB.^18^ Addition of the ATR inhibitor led to the blockade of neutrophil biogenesis that we did not observe with oxaliplatin alone. Yet, the precise ATR function during hematopoiesis remains to be elucidated. In fully differentiated neutrophils and monocytes, the DNA repair machinery including the ATR pathway is compromised.^37^ However, a functional DNA damage response is required in hematopoietic stem cells for normal bone marrow colonization and blood cell biogenesis (for review see^38 39^). Although neutrophil infiltration has been associated with ICB resistance,^40^ here we demonstrated that neutrophil depletion did not recapitulate the Vox effect because it failed to increase the anti-PD-1 antibody efficacy in CRC-PM.

Moreover, we observed the emergence of an Ly-6C^+^PD-1^+^CD8^+^ T-cell population in blood and spleen of mice treated with Vox+anti-PD-1 immunotherapy. This population included a strong proportion of tumor antigen-specific T cells and of BCL6^+^CD122^+^ T cells that might share functional properties with stem-like CD8^+^ T cells and could explain why the Vox+anti-PD-1 antibody combination offers protection against tumor rechallenge. Indeed, stem-like CD8^+^ T cells were first described in cancer models^3341^,^43^ in which they constitute a reservoir in tumor-draining lymph nodes to support the antitumor immunity. These cells can rapidly regenerate a pool of cytotoxic effector cells and accumulate in the mouse spleen in the context of influenza virus infection in the lung.^44^ Furthermore, stem-like CD8^+^ T cells can be identified based on their expression of PD-1, CD122 and TCF1/7, and their induction requires BCL6 expression. In our study, we identified a high proportion of CD122^+^BCL6^+^ cells among Ly-6C^+^PD-1^+^ CD8^+^ T cells in spleen, suggesting that Vox+anti-PD-1 immunotherapy might favor the expansion of this stem-like CD8^+^ T-cell subpopulation.^45^ However, in this study, we failed to purify a large number of these Ly6C^+^PD-1^+^ CD8 T cells from blood or spleen, thus it was impossible to transplant these cells into naïve mice and evaluate their ability to confer immune protection against tumor challenge. The functional demonstration of the critical role of stem-like CD8 T cell in cancer immunity comes from mouse model of melanoma in TCF-7-DTR mice. In these mice, stem-like CD8 T cells depletion using diphtheria toxin abolished the therapeutic effect of ICB plus gp33 vaccination.^43^ These stem-like CD8 T cells are able to proliferate rapidly in tumors where they give rise to tissue resident memory cells displaying antitumor activity. In our study, we observed similarities between Ly6C+PD-1+ T cell and stem-like CD8 T cells, but even if we showed that these cells express CD122, Bcl6 and contain a strong proportion of tumor-specific clones, the functional demonstration of their role in disease control and protection against rechallenge is still missing.

Our work and a recent publication^18^ strongly suggest that ATR inhibition has a direct effect on T cells. However, the molecular mechanism that might favor the emergence of stem-like CD8^+^ T cells remains to be elucidated.

The Vox+anti-PD-1 antibody combination is particularly attractive in the context of ICB-resistant and chemotherapy-resistant disease. Indeed, we propose that ATR inhibition could directly act on T cells potentially driving them towards a stem-like phenotype while simultaneously sensitizing cancer cells to oxaliplatin. This dual action could promote an environment in which anti-PD-1 immunotherapy is more effective. Although these hypotheses need to be experimentally validated. To do so, the two main issues that need to be addressed will be (1) to improve multidrug side effect management and (2) to propose companion test that might help identify patients for whom ATR inhibition will be advantageous.

The clinical trials evaluating ATR inhibitor+anti-PD-1 combination did not report obvious overlapping toxicities.^20 21 46^ Given that oxaliplatin induces hematological and neurological toxicities, and ATR inhibitors mainly hematological toxicities (anemia, myelosuppression), a mitigation of the toxicity (anemia, hematological toxicities, myelosuppression) can be expected when we combine oxaliplatin to ATR inhibitor. To prevent these toxic effects, several strategies can be proposed: (1) a treatment schedule with ATRi ON/OFF, as already performed in preclinical and clinical studies,^16 18 47^ (2) using the treatment locally rather than systemically, as in the case of pressurized intraperitoneal aerosol chemotherapy treatment of peritoneal metastases, and (3) creating an antibody drug conjugate armed with ATRi, which would target a specific tumor cell antigen to deliver the ATR inhibitor locally into the tumor microenvironment.

As a companion test, we propose to measure peripheral blood stem-like CD8 T cells as well as neutrophil number in patients’ blood. Stem-like CD8 T cells can be identified based on their CD58^+^CD95^+^TCF-1^+^PD-1^+^ phenotype. According to our manuscript, the quantity of circulating stem-like CD8 T cells should increase in patients responding to Vox treatment. On the other hand, monitoring neutrophil number will provide important information on VOX toxicity and could help adjust doses of ATR-inhibitor to maximize Vox benefit.

At the end, our work positions Vox+anti-PD-1 antibodies as a new combination therapy that could increase survival in patients with CRC-PM, including those with treatment resistance.

## supplementary material

10.1136/jitc-2024-010791 online supplemental file 1

10.1136/jitc-2024-010791 online supplemental file 2

## Data Availability

Data are available upon reasonable request.
