# Supplementary material for: Oxaliplatin, ATR inhibitor and anti-PD-1 antibody combination therapy controls colon carcinoma growth, induces local and systemic changes in the immune compartment, and protects against tumor rechallenge in mice
Source: J Immunother Cancer. 2025 Mar 26;13(3):e010791. doi: 10.1136/jitc-2024-010791 (PMC11950992; doi:10.1136/jitc-2024-010791)
Supplement: online supplemental file 1 [file jitc-13-3-s001.pdf]

Oxaliplatin, ATR inhibitor and anti-PD-1 antibody combination therapy controls colon carcinoma growth, induces local and systemic changes in the immune compartment, and protects against tumor rechallenge in mice

Alexandra Fauvre*, Chiara Ursino*, Veronique Garambois, Elodie Culerier, Lois-Antoine Milazzo, Nadia Vezzio-Vié, L Jeanson, Candice Marchive, AF Andrade, Eve Combes, Salima Atis, G Lossaint, F Quenet, Henri-Alexandre Michaud, Lakhdar Khellaf, I Corbeau, Diego Tosi, Nadine Houede, Nathalie Bonnefoy, Sgarbura Olivia, Céline Gongora#, and Julien Faget#.

* These authors contributed equally as first author

\# These authors contributed equally as principal investigator and corresponding author.

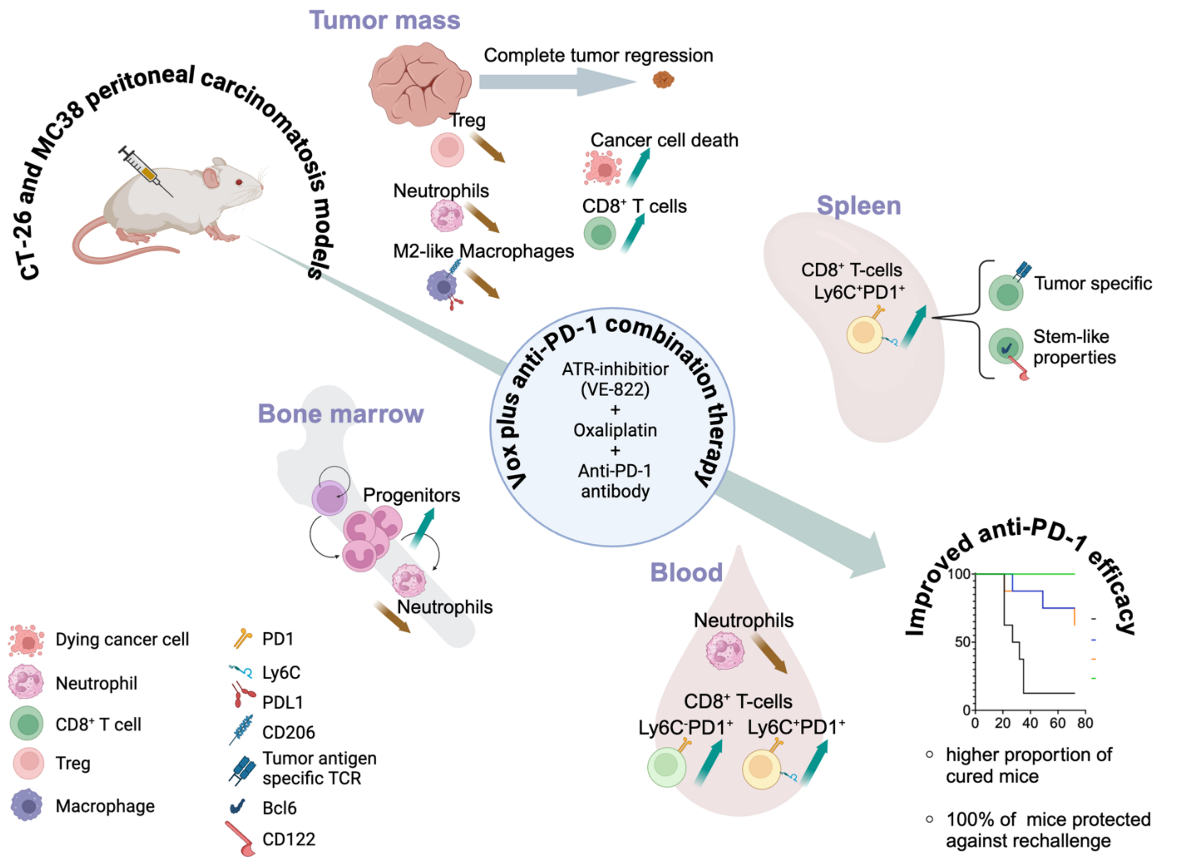

- ⇨ Combination of VE-822 + oxaliplatin (Vox) and anti-PD-1 antibody completely cured CRC-bearing mice and protected them from tumor rechallenge.
- ⇨ Vox deeply altered neutrophil biogenesis and induced the accumulation of Ly6C$^+$PD-1$^+$CD8$^+$ T cells in the mouse blood and spleen.
- ⇨ Vox + anti-PD-1 immunotherapy could overcome oxaliplatin resistance.
