# Supplementary material for: Oxaliplatin, ATR inhibitor and anti-PD-1 antibody combination therapy controls colon carcinoma growth, induces local and systemic changes in the immune compartment, and protects against tumor rechallenge in mice
Source: J Immunother Cancer. 2025 Mar 26;13(3):e010791. doi: 10.1136/jitc-2024-010791 (PMC11950992; doi:10.1136/jitc-2024-010791)
Supplement: online supplemental file 2 [file jitc-13-3-s002.docx]

**Oxaliplatin, ATR inhibitor and anti-PD-1 antibody combination therapy controls colon carcinoma growth, induces local and systemic changes in the immune compartment, and protects against tumor rechallenge in mice**

Alexandra Fauvre*, Chiara Ursino*, Veronique Garambois, Elodie Culerier, Lois-Antoine Milazzo, Nadia Vezzio-Vié, L Jeanson, Candice Marchive, AF Andrade, Eve Combes, Salima Atis, G Lossaint, F Quenet, Henri-Alexandre Michaud, Lakhdar Khellaf, I Corbeau, Diego Tosi, Nadine Houede, Nathalie Bonnefoy, Sgarbura Olivia, Céline Gongora#, and Julien Faget#.

* These authors contributed equally as first author

# These authors contributed equally as principal investigator and corresponding author.

**Supplemental materials**

Supplemental Material and methods

Supplemental figures and figure legends

**Supplemental Material and methods**

**Drugs and antibodies**

Oxaliplatin was from the Montpellier Institute of Cancer pharmacy. VE-822 was from Selleckchem, the anti-PD-1 monoclonal antibody (mAb) (CD279) for injection in mice was from BioXCell.

Primary antibodies used for western blotting were against: PD-L1 (CST rabbit mAb #13684), cGAS (D3O8O rabbit mAb #31659), STING (D2P2F rabbit mAb (#13647), TBK1/NAK (E8I3G rabbit mAb #38066), phosphorylated TBK1/NAK (Ser172) (D52C2 XP® rabbit mAb #5483), IRF-3 (D83B9 rabbit mAb #4302), phosphorylated IRF-3 (Ser396) (D6O1M rabbit mAb #29047), NF-κB p65 (D14E12 XP® rabbit mAb #8242), phosphorylated NF-κB p105 (Ser932) (18E6 rabbit mAb #4806), and GAPDH (14C10 rabbit mAb #2118), from Cell Signaling Technology. IRDye 680RD goat anti-mouse IgG (#925-68070), IRDye 680RD goat anti-rabbit IgG (#925-68071), IRDye 800CW goat anti-mouse IgG (#925-32210), and IRDye 800CW goat anti-rabbit IgG (#925-32211) (all from LI-COR Biosciences) were used as secondary antibodies.

**Cell culture**

Certified human HCT116 cells were obtained from ATCC (CCL-247) and murine CT26 cells were kindly provided by Nathalie Bonnefoy. Cells were cultured in RPMI1640 supplemented with 10% fetal calf serum and 2mmol/L L-glutamine at 37^o^C in a humidified atmosphere with 5% CO_2_. The murine CRC cell line MC38, provided by N. Bonnefoy, was grown in DMEM with 10% fetal calf serum and 1mM sodium pyruvate at 37°C in a humidified atmosphere with 5% CO2. The oxaliplatin-resistant clone HCT116-R1 was obtained as previously described^23^. Briefly, oxaliplatin-sensitive parental HCT116 cells were grown in the presence of 5-10μM oxaliplatin and cloned to obtain the resistant HCT116-R1 cell line. All cell lines were authenticated by short tandem repeat profiling (LGC Standards and Eurofins Genomics). All experiments were performed with mycoplasma-free cells. No antibiotic was used to avoid any cross-reaction with oxaliplatin and VE-822. CT26 and MC38 cells were transfected with a plasmid expressing luciferase for in vivo bioluminescence detection and GFP.

**Animals**

Female BALB/c and C57BL/6J mice (5-6-week-old) (Charles River Laboratories, Saint-Germain-Nuelles, France) were housed in filter-topped cages (5 mice per cage) in clean, non-sterile, standardized conditions (temperature 20–24 °C, relative humidity 50–60%, 12 h light/12 h dark cycle). They were fed a standard laboratory diet and tap water *ad libitum*. Mouse experiments were performed in compliance with the French regulations and ethical guidelines for experimental animal studies in an accredited establishment (Agreement No. #31135-2021042212479661/Referral n°02095, Animal Protection Committee, French Ministry of Agriculture).

**In vivo studies:** All in vivo experiments were performed in compliance with the French regulations and ethical guidelines for experimental animal studies in an accredited establishment. Ethical approvals were obtained by the local ethics committee [Ethics Committee approved by the French Ministry, animal facility approval C34-172-27, personal approval (Céline Gongora) 34.142, and protocol approval APAFIS#25403 and APAFIS #42660.

**Subcutaneous grafts**: MC38 cells (5x10^5^) were injected subcutaneously in the left flank of 8-week-old female C57/Bl6 mice (Charles River Laboratories, France) (n=6-8). Tumors were detected by palpation and measured with a caliper weekly. Mice were euthanized when the tumor volume reached 1500 mm^3^.

***Orthotopic model***

The CT26-Luc cell suspension (1.0 × 10^5^ cells (first inoculation) or 2.0 × 10^5^ (rechallenge) cells in 100 μL of serum-free RPMI media) or MC38-Luc cell (5.0 × 10^4^ cells (first inoculation) or 1.0 × 10^5^ (rechallenge) in 100 μL of PBS) was injected into the peritoneum in the left iliac fossa of mice using a 25-gauge needle. When the bioluminescence reached a score between 10^7 and 10^8 (approximately 5 days after injection), tumor-bearing mice were randomized. Tumor-bearing mice were treated on day +7 for survival studies and day +12 for immunophenotyping with Ve-822 (60mg/kg diluted in NaCl+TPGS) by gavage, oxaliplatin (5mg/kg diluted in Ppi water) or/and anti-PD1(10mg/mL diluted in NaCl) or saline solution by intraperitoneal injection. Ve-822 and anti-PD1 was injected twice a week, and oxaliplatin once every two weeks. Mice were monitored by bioluminescence once a week. Survival curves were doing by the Kaplan-Meier method. For some experiment, cured mice were subjected to rechallenge two month after treatment interruption. For rechallenge experimented we injected twice the number cancer cells used for primary challenge.

**Flow cytometry**

Tumor, spleen, femur (for bone marrow) and blood samples were collected from mice. Tumors were dissected, minced using rounded scissors, and digested in DNase I (150 U/mL, Sigma, D5025-15KU) and collagenase (1 mg/mL, Sigma, C1889-50MG) in DMEM using a gentleMACS Dissociator (Milteny Biotec, 130-093-235). Cells were filtered through 70µm filters (Falcon, 352350), washed and resuspended in 20mg/100µL of FACS buffer (PBS/2% BSA/0.5mM EDTA). Bone marrow was flushed with a needle and syringe from Henke Sass Wolf. Spleens were weighed, crushed and filtered through 40 µm cell strainers (Falcon, 352340) using FACS buffer. Red blood cells were lysed (Miltenyi Biotec) for 10 min.

Cells were incubated with Precision Count Beads (BioLegend, 424902) and stained with fluorescently labeled antibodies in FACS Buffer at 4°C for 15 min. Cells were permeabilized using the Fixation/Permeabilization kit from eBioscience (00-5523-00) for intracellular staining. Cell viability was assessed by staining with the Viakrom dye followed by fixing in 1% paraformaldehyde. Data were acquired on a Cytoflex LX flow cytometer (Beckman Coulter) and analyzed with FlowJo® (Tree Star). The complete list of the used antibodies is in table S1. The gating strategy is presented in online supplemental figures 2A, 3B and figure 7B.

**Immunohistochemistry (IHC) analyses**

For IHC, PM nodules were grouped together and fixed in 10% neutral buffered formalin for 24 h, dehydrated, embedded in paraffin, and cut into 3-µm-thick sections, mounted on slides, and dried at 37°C overnight.

IHC was performed on a VENTANA Discovery Ultra automated staining instrument (Ventana Medical Systems) using VENTANA reagents, according to the manufacturer’s instructions. For 2-plex FOXP3-CD8 IHC staining, slides were de-paraffinized and epitope retrieval was performed with the CC1 solution (cat# 950-124) at 95°C for 24 min. Endogenous peroxidases were blocked with Discovery Inhibitor (cat# 760-4840). The primary antibody against FOXP3 (Ozyme, cat#12653S, 1:200) was added at 37°C for 60 min followed by signal enhancement using the OmniMap anti-rabbit detection kit (cat# 05266548001) for 16 min and incubation with DAB (cat# 05266645001). After stripping in CC2 buffer at 100°C for 8 min, the rabbit anti-CD8 antibody (Cell Signaling, cat#98941, 1:100) was added at 37°C for 60 min followed by signal enhancement using the OmniMap anti-rabbit detection kit (cat# 05266548001) and a purple chromogen (cat# 07053983001). Sections were counterstained with hematoxylin II (cat# 790-2208) for 8 min, followed by Bluing reagent (cat# 760-2037) for 4 min. Slides were dehydrated in a Leica autostainer and cover slip and Pertex mounting medium added by a CTM6 coverslipper (Microm).

Slides were digitalized with a Hamamatsu NanoZoomer 2.0-HT scanner or with a PANNORAMIC Midi II slide scanner (3DHISTECH). QuPath version 0.5.1 (Bankhead et al., 2017) was used to quantify Treg and CD8^+^ T cells. A script based on pixel classification was used to identify FOXP3^+^ and CD8^+^ cells. The percentage area occupied by these different cell populations was calculated as the ratio positive cells to all cells.

**Western blot analysis**

After counting, 10,000 cells/µL were washed with PBS and directly lysed in Laemmli buffer (4% SDS, 20% glycerol, 1% 2-β mercaptoethanol, 0.004% bromophenol blue, 0.125 M Tris HCL) in the presence of benzonase (25 units/ mL). After denaturation at 95 °C for 5 min, protein extracts were separated on SDS-PAGE polyacrylamide gels and transferred to nitrocellulose membranes (0.45μm pore size, Amersham Pharmacia Biotech, Uppsala, AB, Sweden). Then, membranes were blocked in PBS/0.1% Tween-20/5% milk at room temperature for 1 h and incubated with primary antibodies at 4 °C under gentle agitation overnight. After three washes with PBS/0.1% Tween-20, membranes were incubated with the relevant anti-species secondary antibody coupled to a fluorochrome at room temperature for 1 h. Immunoreactions were revealed by fluorescence and quantified with a LI-COR Imager (LI-COR Biosciences). Protein expression levels were normalized to the loading control or to the total protein loaded using Revert™700 Total Protein Stain.

**RT-qPCR analysis**

**Cells**: For RNA extraction, cells were seeded in 6-well plates (106 cells per well). The day after, cells were incubated with the indicated drugs and total RNA was extracted using the Quick-RNA Miniprep Kit (Zymo Research, #R1055), according to the manufacturer’s instructions. RNA quantity and quality were determined using a Nanodrop 2000 (ThermoScientific, Waltham, MA, USA) (260/280 nm absorbance ratio). cDNAs were obtained by reverse transcription performed using the SuperScript III kit (Thermo Fisher). Briefly, 500ng of total RNA was added to the reverse transcription mix containing 4 μL of buffer 5X, 1 μL of 10 mM dNTP mix, 0.5 μL of random primer solution, 1µL of DTT 0.1mM, and 1 µL of SuperScript III (200 U/μL) and adjusted to 20 μL final volume with RNase-free water.

**Tumor samples:** Total RNA was extracted using the phenol-chloroform method and the TRI reagent (Merk/Sigma-Aldrich Ref: T9424) according to the manufacturer’s instructions. 1µg of RNA was reverse transcribed using PrimSTAR SuperScript III (ThermoFisher Ref:1808005).

**qPCR analysis**: 10 ng of cDNA was analyzed by qPCR in duplicate using the ONEGreen FAST qPCR Premix (OZYME Ref: OZYA008- 40/OZYA008-200XL) and a Light Cycler 480 II (Roche) instrument. Expression levels were determined using the ΔΔCt method and were normalized to housekeeping genes (*GAPDH, HPRT, RPL0* or *TBP-1*). The primers sequences are in table S2.

**Statistics**

Data were expressed as the mean ± SEM of values from at least three experiments. The relationship between tumor growth and treatment was analyzed using a linear mixed regression model. The fixed part of the model included the number of days post-graft and treatment group; interaction terms were also evaluated. Random intercepts and random slopes were included to take into account the time effect. The model coefficients were estimated by maximum likelihood. A survival analysis was performed and the event considered was a tumor volume of 1500 mm^3^. Survival rates were estimated using the Kaplan Meier method and survival curves were compared with the log rank test. Statistical significance was set at *p*<0.05. Statistical analyses were done with STATA 16 (Stata Corporation, College Station, TX).

**Table S1** Antibodies for flow cytometry analyses

| **Name/target** | **Labeling** | **Company** | **Reference** |
| --- | --- | --- | --- |
| Beads |  | BioLegend | 424902 |
| Viakrom | IR808 | BeckmaCoulter | C36628 |
| Dextramer | PE | Immudex | JE03822 PE 50 |
| CD45 | BV750 | BioLegend | 103157 |
| CD4 | BUV495 | BD Biosciences | 741134 |
| CD8 | BUV395 | BD Biosciences | 563786 |
| CD206 | FITC | BioLegend | 141703 |
| CD206 | APC | BioLegend | 141708 |
| CD103 | BV510 | BioLegend | 121423 |
| Ly-6G | AF700 | BioLegend | 127622 |
| Ly-6C | PeCy7 | BioLegend | 128018 |
| Ly-6C | PE | Milteny Biotec | 130-117-522 |
| CD11b | BV510 | BioLegend | 101263 |
| CD11c | BV421 | BioLegend | 117343 |
| PD-1 | BV510 | BioLegend | 135241 |
| PD-1 | Pedzz | BioLegend | 135228 |
| PD-L1 | Pedzz | BioLegend | 124324 |
| F4/80 | BV605 | BioLegend | 123133 |
| B220 | FITC | BioLegend | 103206 |
| CD3 | PE-Vio770 | Milteny Biotec | 130-116-530 |
| CD3 | PE | BD Biosciences | 555275 |
| CD3 | BV421 | BioLegend | 155617 |
| TER-119 | Percp | BioLegend | 116226 |
| NKp46 | BV605 | BioLegend | 137619 |
| Gr-1 | AF700 | BioLegend | 108422 |
| CD117/cKit | PE | BD Biosciences | 553355 |
| CD16/32 | BV421 | BioLegend | 101331 |
| CD34 | PE | BioLegend | 119308 |
| SCA1 | APC | Milteny Biotec | 130-123-848 |
| CD122 | PE-Cy7 | BioLegend | 123215 |
| CXCR3 | BV510 | BioLegend | 126538 |
| EOMES | PE | BioLegend | 157706 |
| CD62L | BV605 | BioLegend | 104438 |
| BCL6 | APC | Invitrogen | 17-5453-82 |
| CD19 | AF488 | BioLegend | 115521 |
| CD44 | APC-F750 | BioLegend | 156004 |
| CD25 | APC_Fire_750 | BioLegend | 102053 |

**Table S2**: Primers used for RT-qPCR (mouse genes).

| Gene | **Forward 5'->3'** | **Reverse 5'->3'** |
| --- | --- | --- |
| Bcl6 | CTCCTCAGAGAAACGGCAGTCA | CAGAGATGTGCCTCCATACTGC |
| Ccl2 | TTAAAAACCTGGATCGGAACCAA | GCATTAGCTTCAGATTTACGGGT |
| Ccl5 | CCCTCACCATCATCCTCACT | CCTTCGAGTGACAAACACGA |
| Cd3 | ATGCGGTGGAACACTTTCTGG | GCACGTCAACTCTACACTGGT |
| Ptprc | ATGGTCCTCTGAATAAAGCCCA | TCA GCA CTA TTG GTA GGC TCC |
| Cxcl1 | GCTGGGATTCACCTCAAGAA | AGGTGCCATCAGAGCAGTCT |
| Cxcl10 | CCAAGTGCTGCCGTCATTTTC | GGCTCGCAGGGATGATTTCAA |
| Cxcl2 | CCAACCACCAGGCTACAG | GCGTCACACTCAAGCTCTG |
| Cxcl5 | CCGCTGGCATTTCTGTTGCTG T | CAGGGATCACCTCCAAATTAGCG |
| Gzmb | CAGGAGAAGACCCAGCAAGTCA | CTCACAGCTCTAGTCCTCTTGG |
| Ifng | ATGAACGCTACACACTGCATC | CCATCCTTTTGCCAGTTCCTC |
| Ifnb | CAGCTCCAAGAAAGGACGAAC | GGCAGTGTAACTCTTCTGCAT |
| Il15 | AAAGGCATTCCAGGACACAC | CCAACCCCAGCTAACAGAAA |
| Il6 | TTCCATCCAGTTGCCTTCTTG | GGGAGTGGTATCCTCTGTGAAGTC |
| Isg15 | CATCCTGGTGAGGAACGAAAGG | CTCAGCCAGAACTGGTCTTCGT |
| Lef1 | ACTGTCAGGCGACACTTCCATG | GTGCTCCTGTTTGACCTGAGGT |
| Pd1 | CGTCCCTCAGTCAAGAGGAG | GTCCCTAGAAGTGCCCAACA |
| Tcf1/7 | CCTGCGGATATAGACAGCACTTC | TGTCCAGGTACACCAGATCCCA |
| Tnf | CTGAACTTCGGGGTGATCGG | GGCTTGTCACTCGAATTTTGAG |

**Supplemental figure legends and table S3**

**Figure S1: Immune response gene expression in CT26 cells after incubation with Vox**

A) Histograms showing the relative mRNA expression of the indicated genes in CT26 cells incubated with PBS (Ctr), oxaliplatin (Ox) VE-822 (Ve) or Vox for 24 hours. *p*=0.05; ***p*=0.01; ****p*=0.001 (one-way ANOVA).

**Figure S2: Vox treatment quantitatively and qualitatively remodels the tumor immune compartment**

A) Plots showing the quantitative flow cytometry gating strategy used in figure 4. B) Spearman correlations for the quantitative data on immune cells in all samples. C and D) Histograms showing the percentage of the indicated immune cell types relative to all tumor-infiltrated immune cells from the same samples described in figure 4E. E) Histograms showing the relative expression of *Ptprc* (CD45) in the same samples as in figure 4F. **p*=0.05; ***p*=0.01; ****p*=0.001; *****p*=0.0001 (one-way ANOVA).

**Figure S3: Neutrophil biogenesis is altered upon Vox treatment**

A) Histograms showing the relative mRNA expression of the indicated genes in the indicated FACS-sorted cell types from the same samples as in figure 4F. **p*=0.05; ****p*=0.001 (one-way ANOVA). B) Plots showing the quantitative flow cytometry gating strategy used to identify LSK (Lin^-^CD11b^-^Gr-1^-^c-Kit^+^SCA1^+^), LK (Lin^-^CD11b^-^Gr-1^-^c-Kit^+^SCA1^-^), GMP (Lin^-^CD11b^-^Gr-1^-^c-Kit^+^SCA1^-^CD16/32^+^CD34^+^), Gr-1^low^ cells (CD11b^+^Gr-1^Low^) and mature neutrophils (CD11b^hi^Gr-1^hi^). C) Proposed model that recapitulates Vox effect on neutrophil biogenesis and infiltration in PM lesions. TAN, tumor-associated neutrophils. D) Plots showing the flow cytometry gating strategy used to monitor neutrophil number in blood samples from mice treated with isotype IgG control, anti-Ly-6G antibody, and Vox.

**Figure S4: Neutrophil depletion does not recapitulate Vox effect on Ly-6C^+^PD-1^+^ T cell accumulation**

A, B) Number per mg of tumor A) and percentage of the indicated cell sub-populations among CD8^+^ T cells of GFP-Luc-CT26 PM samples from mice not treated (Ctr, n=7) or treated with the anti-PD-1 antibody (n=8) or Vox + anti-PD-1 antibody (n=9). C) Histograms showing the number per ml of blood (left and middle) and the percentage among CD8^+^ T cells (right) of the indicated immune cell populations in the blood of GFP-Luc-CT26 PM mice untreated (NT n=5) or treated with the anti-Ly-6G antibody (n=6), anti-Ly-6G + anti-PD-1 antibodies (n=6), anti-Ly6G antibody + Vox (n=6), or anti-Ly-6G antibody + Vox + anti-PD-1 antibody (n=6). D) Relative mRNA expression of the indicated genes in the indicated CD8^+^ T cell sub-populations from blood of the same mice as in figure 6E (n=5 per condition). **p*=0.05; ***p*=0.01; ****p*=0.001 (one-way ANOVA).

**Figure S5: Stem cell-like markers in spleen CD8^+^ T cells from mice treated with Vox + anti-PD-1 antibody**

A-B) Graphs showing the relative mRNA expression of the indicated genes in the indicated CD8^+^ T-cell subpopulations from spleen (A) and blood (B) of GFP-Luc-CT26 PM mice treated with Vox + anti-PD-1 antibodies (n=5 pools of 4 mice); ***p*=0.01 (one-way ANOVA). C and D) Plots showing the flow cytometry gating strategy used to monitor BCL6 and CD122 expression on CD8^+^ T-cell subpopulations in spleen from GFP-Luc-CT26 PM mice treated with anti-PD-1 antibody (C) and Vox + anti-PD-1 antibody (D).

**Table S3: Summary of current clinical trials involving ATR inhibitors**
